# The use of an ultrasonic cement removal device in revision hip and knee arthroplasty—A matched case‐control study

**DOI:** 10.1002/jeo2.70171

**Published:** 2025-04-15

**Authors:** Marco Haertlé, Lars‐René Tücking, Alexander Derksen, Magnus Reulbach, Eike Jakubowitz, Henning Windhagen

**Affiliations:** ^1^ Department of Orthopaedic Surgery Hannover Medical School Hannover Germany; ^2^ Department of Orthopaedic Surgery Laboratory for Biomechanics and Biomaterials (LBB), Hannover Medical School Hannover Germany

**Keywords:** cemented arthroplasty, hip revision surgery, knee revision surgery, ultrasonic cement removal

## Abstract

**Purpose:**

The objective of this case series was to investigate the effect of ultrasound‐guided cement removal devices on operating time and patient safety in the revision of cemented knee and hip arthroplasties.

**Methods:**

A total of 11 cases were examined in which ultrasound‐guided cement extraction was utilised for implant removal. The primary endpoint of the study was the duration of the surgery. Additionally, the cohort was analysed for the occurrence of intraoperative fractures or postoperative ossification. Postoperative laboratory dynamics of haemoglobin and C‐reactive protein levels were also investigated. A matched group of 11 patients who underwent revision arthroplasty using conventional techniques to remove bone cement served as the reference group.

**Results:**

Ultrasound‐guided removal of cement from the medullary canal was associated with a significantly longer operation time (187.19 min ± 54.4 vs. 121.91 min ± 43.5, (*p* = 0.0026). Furthermore, there was a significant decrease in haemoglobin drop relative to baseline haemoglobin levels when ultrasound‐guided tools were employed for cement removal (2.36 g/dL ± 1.9 vs. 4.54 g/dL ± 1.9, *p* = 0.0015). Moreover, an intraoperative fracture complication of the femoral shaft was observed when the cement was removed using an ultrasonic cement stripper.

**Conclusion:**

A comparison between the two groups reveals a significant increase in surgical duration when cement removal was performed using ultrasound‐guided technique. Simultaneously, the use of an ultrasound‐assisted system for cement removal did not mitigate the risk of intraoperative bone perforation. Based on the data presented in this study, the authors cannot conclude that the use of ultrasound‐guided devices for the removal of cement residues from the medullary canal during revision surgery is superior to conventional techniques.

**Level of Evidence:**

Level IV.

**Abbreviations**
ANOVA: analysis of variance
MCR: manual cement removal
THA: total hip arthroplasty
TKA: total knee arthroplasty
UCR: ultrasonic cement removal

## INTRODUCTION

In recent decades, the frequency of knee and hip implant procedures has consistently increased. Concurrently, the average age of patients undergoing joint replacement surgeries has also risen steadily. Given the escalating number of hip and knee prostheses implantations and the increasing age of patients, it is anticipated that the incidence of revision surgeries for knee and hip implants will continue to rise in the coming years [17, 19, 20]. It is anticipated that, in addition to the health burden, there will be substantial financial pressure on healthcare systems to manage these patients, emphasising the significance and necessity of innovation and research in this surgical field [4].

The most prevalent indications for revision surgery in knee and hip arthroplasty are periprosthetic joint infections, periprosthetic fractures, and aseptic loosening of implants [1, 23, 26]. Knee and hip implants can be secured in the patient's bone through various methods. In addition to cementless implantation of endoprostheses, which relies on press‐fit anchoring of the implant to the bone, it is also feasible to secure implants using bone cement. Particularly in patients with compromised bone structure, such as in cases of significant osteoporosis, the utilisation of bone cement appears to be superior compared to cementless implant fixation [9]. Irrespective of bone quality, the majority of knee prostheses are currently implanted using bone cement [18].

Bone cement typically comprises a solid powder component (polymethyl methacrylate) and a liquid stabiliser. Through an exothermic reaction, this chemical mixture solidifies in the interface between the bone and the implant, thereby ensuring the stability of the prosthesis [25]. In the event of revision surgery necessitating the removal of the implant components, the administered cement presents a significant surgical challenge, as a rigid bond has formed between the bone and the cement surface. Cortical perforation may occur when attempting to breach the cement mantle. Furthermore, the complete removal of the bone cement in the medullary cavity poses a surgical and temporal challenge due to the fragmentation characteristics of the solidified bone cement. Prolonged cement removal generally increases the risk of infection for the patient due to the extended duration of the surgery [13, 16, 21].

In response to this surgical challenge in cement removal, ultrasound‐based cement removal systems have been introduced. These systems utilise an oscillating ultrasonic drill to soften the cement, thus facilitating complication‐free cement extraction. The primary advantage of employing ultrasonic devices for cement removal is purported to lie in the selective softening of the bone cement while preserving the bone tissue [12]. However, the extent to which the utilisation of such systems contributes to patient safety remains unresolved to date.

## METHODS

### Patients

This study analysed a cohort of 11 patients who underwent arthroplasty revision surgery utilising ultrasonic cement removal between October 2021 and January 2024. The inclusion criterion for this retrospective study was the utilisation of an ultrasound‐guided cement removal system in the context of removal or replacement of hip and knee implants.

The surgical procedures in this series were performed by four senior orthopaedic surgeons, each possessing experience in over 100 arthroplasty procedures. A control group of 11 patients who underwent arthroplasty revision surgery with manual removal of inserted cement was matched according to age and type of revision surgery for comparative analysis.

This study included three categories of revision surgery. First, the explantation of total hip arthroplasties (THA) and total knee arthroplasties (TKA) due to septic implant failure necessitating a two‐stage revision was included. Furthermore, aseptic single‐stage revisions of cemented TKA were incorporated in the study. Patients who underwent single‐ or two‐stage revision surgery of non‐cemented implants were excluded from this investigation. Additionally, patients in whom a standard bicondylar TKA without femoral and tibial stem had been removed or replaced were also excluded from the study. Only patients who had undergone revision surgery with cemented femoral and tibial stems were deemed eligible for inclusion. Table 1 presents the demographic characteristics of the patients.

**Table 1:** Descriptive data manual cement removal (MCR) versus ultrasonic cement removal (UCR) for total hip (THA) and total knee arthroplasty (TKA).

Number of patients	MCR	UCR	*p*‐value
Type of revision surgery	11/22	11/22	
Explantation cemented THA	1/11	1/11	
Explantation cemented TKA	4/11	4/11	
One‐stage revision cemented TKA	6/11	6/11	
Ø Age (years)	68.82 ± 13.17	69.00 ± 13.51	0.4802
Sex			
Male	4/11 (36.26%)	6/11 (54.55%)	
	7/11 (63.64%)	5/11 (45.45%)	0.3918
Body mass index (kg/m^2^)	29.72 ± 7.77	31.57 ± 5.7	0.2757
ASA* risk classification			
I	1/11	0/11	
II	6/11	9/11	
II	4/11	3/11	0.4269
(*American Society of Anesthesiologists)			

### Surgical technique

**Figure 1:**
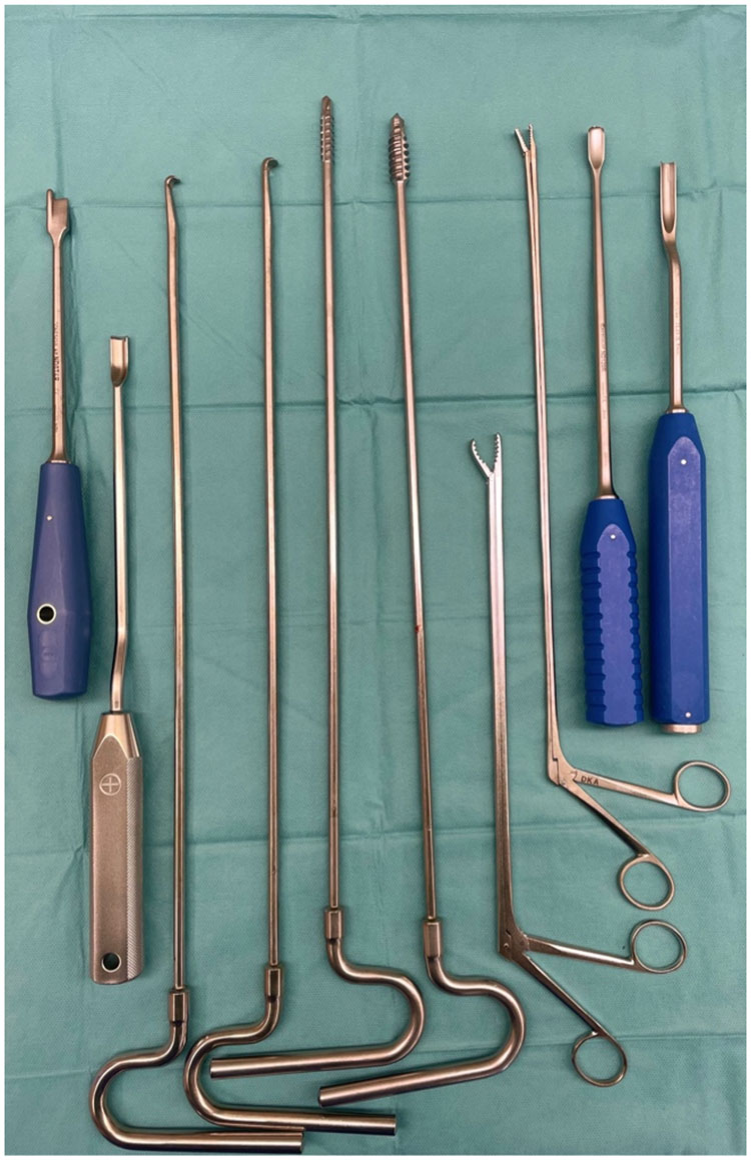
A set of specially designed cement removal chisels and drills typically used to crack the residual cement plug or create a gap at the cement–bone interface, facilitating the extraction of cement from the medullary canal.

Conventional removal of cement is performed utilising osteotomes of varying sizes or through spot drilling using K‐wires along the bone‐implant interface. Following complete extraction of the implant, residual cement within the intramedullary cavity is removed using diverse chisels, drills, and rongeurs (Figure 1). In certain instances, an osteotomy of the bone may be necessitated to fully extract the remaining cement [14]. To achieve a more atraumatic removal of cement from the osseous interface, ultrasonic devices were introduced for this precise procedure [22]. The fundamental principle of ultrasound‐guided cement removal is that repetitive friction generates stress waves exceeding 16 kHz, thereby readily elevating the temperature of bone cement due to its energy absorption capacity and low thermal conductivity.

The required oscillating forces are generated by the friction between the ultrasonic device tip and the cement mantle, resulting in the vaporisation or liquefaction of the bone cement without compromising the bone matrix [7]. Based on this technique, specialised devices have been developed for the atraumatic removal of cement residues utilising ultrasound technology in revision arthroplasty. The OSCAR Pro™ device (Orthofix, United States of America, Lewisville, TX) was used in this study. Titanium cement scrapers and piercers with diameters ranging from 6 to 10 mm were utilised as probes. The cement removal process initiates with the loosening of the most proximal part of the cement socket using a small‐calibre piercer, creating an opening in the bone–cement interface. Subsequently, the probe is inserted along the implant surface and advanced downwards until the implant is liberated from the cementation and can be easily extracted. In instances where a residual cement plug is present, a central opening is created in the cement body using a large‐calibre piercer. Once the ultrasonic device tip has penetrated the cement plug, the cement resolidifies behind and around the probe. The cement plug can then be extracted en bloc in a retrograde manner from the intramedullary space by carefully withdrawing the device or by dislodging the cement plug with an ejector.

### Variables and endpoints

The surgical time required to completely remove the implanted prosthesis was defined as the primary endpoint of this study. The duration of the surgery was obtained from the electronic documentation. As secondary outcome measures, we assessed the Δ haemoglobin value, the Δ CRP value, the intraoperative blood loss, the incidence of fracture or infection complications and the occurrence of ossifications. The results of the blood tests were extracted from the electronic patient file. Blood samples were taken on a standardised basis on the 1st, 3rd, and 5th postoperative days. Subsequently, blood parameters were determined three times a week. The occurrence of intraoperative fractures and the development of ossification was investigated using intraoperative and postoperative X‐ray imaging.

### Statistical analysis

The data were recorded in tabular form, and the continuous variables were expressed as mean ± standard deviation and range. The significance level between the mean values was tested using analysis of variance (ANOVA). There were no missing data for the conduction of this analysis. A *p*‐value of < 0.05 was considered statistically significant. PRISM 10 (GraphPad Software, United States of America, Boston, MA) was used for statistical analysis. The study was conducted in accordance with the Declaration of Helsinki and received approval by the local Ethics committee (11428_BO_K_2024).

## RESULTS

**Figure 2:**
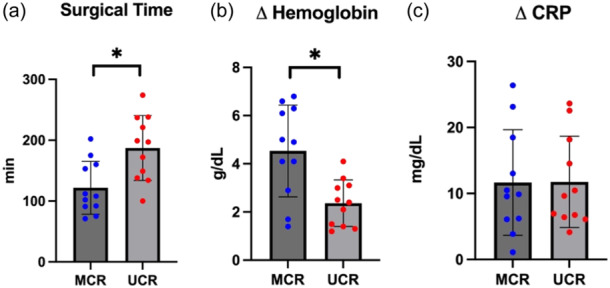
(a) The use of the ultrasonic cement removal device (UCR) was associated with a significantly longer operation time compared to manual cement removal (MCR). (b) Ultrasoundguided cement removal (UCR) was associated with a significant smaller decrease in haemoglobin level relative to the baseline haemoglobin (Δhaemoglobin). (c) The choice of removal technique had no statistically significant influence on the relative change in the postoperative C‐reactive protein (CRP) level.

The study analysed a case series comprising 22 operations involving bone cement removal. In 11 cases, the cement was manually removed using the chisels depicted in Figure 1. In the remaining 11 surgeries, an ultrasonic device was employed for cement remnant removal. Notably, the operative time for manual cement removal (MCR) was 121.91 min ± 43.5 on average, compared to cement removal with ultrasound‐based devices (UCR), which required an average of 187.19 min ± 54.4 (*p* < 0.01) (Figure 2a). Furthermore, the comparison between MCR and UCR in terms of relative reduction in haemoglobin revealed that UCR was associated with a significantly lower Δhaemoglobin of 2.36 g/dL ± 1.9. The MCR technique demonstrated a Δhaemoglobin of 4.54 g/dL ± 1.9 (*p* < 0.01) (Figure 2b). The choice of removal technique did not exert a statistically significant influence on the relative change in postoperative inflammation state, as measured by CRP level (*p* = 0.49) (Figure 2c).

**Figure 3:**
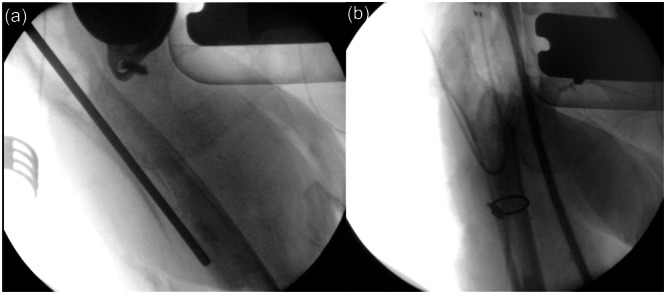
(a) The intraoperative X‐ray shows a perforation of the femoral shaft during ultrasound‐guided cement removal. (b) After complete removal the bone cement, the fracture was stabilised using a cerclage wire in a Girdlestone situation.

In the study cohort of patients in which the manual cement removal technique was employed, no perioperative fractures or postoperative ossification were observed. In the group of patients who underwent surgery utilising the ultrasound‐guided cement removal device, one intraoperative fracture of the femoral shaft, characterised as a via falsa, was documented. The femoral shaft was subsequently stabilised using a cerclage (Figure 3a,b).

## DISCUSSION

We examined 11 patients in whom hip, or knee implants were explanted or replaced using ultrasound‐guided cement removal system. We compared these 11 patients with matched patients in whom manual cement extraction was performed. We found that manual cement extraction during revision surgery was not only associated with a shorter operation time, but also with a lower fracture rate.

Given the time‐consuming and laborious process of removing cement from the medullary canal, one of the objectives of utilising ultrasonic cement removal devices is to minimise the operation time. Reduced operation time also contributes to a decrease in septic complications in the field of joint surgery [15]. Notably, the first significant finding of our study was that the use of ultrasonic cement removal devices significantly prolonged the duration of surgery, thus producing a counter‐intuitive effect. A learning curve effect can be anticipated in this context due to the limited case series of 12 applications of ultrasonic cement removal devices, further compounded by the involvement of four different surgeons. Achieving proficient handling of the ultrasonic cement removal device, minimising complications, and optimising operating time undoubtedly requires additional experience by surgeons. Comparing manual bone cement removal with ultrasound‐guided removal, manual removal is significantly more straightforward. Chisels and osteotomes used in manual removal can rapidly open and extract the cement; however, from our experience, it often results in an uncontrolled dispersion of cement fragments. These fragments can be ejected in multiple directions from the surgical site and may contaminate the surgical field through contact with the face mask. Therefore, a thorough consideration of the balance between the aggressiveness of cement removal and the duration of the operation is essential to avoid septic complications.

Notably, a reduced decrease in haemoglobin level was observed in the cohort treated with ultrasonic cement removal, indicating reduced intraoperative blood loss. Given that accurate assessment of blood loss presents a significant challenge, particularly in retrospective studies, the Δhaemoglobin value serves as an established method for estimating intraoperative blood loss with greater accuracy [6]. Reduced blood loss generally results in a lower risk of periprosthetic infection and facilitates expedited postoperative mobilisation of the patient [10]. A potential explanation for the reduced intraoperative blood loss concurrent with prolonged operation duration may be attributed to the local hyperthermia that develops across the bone during ultrasonic melting of the bone cement, subsequently leading to automatic haemostasis of the endosteal capillary vessels.

The thermal effect of ultrasonic melting of bone cement, which can result in tissue damage extending beyond the bone structure into the soft tissue, has been previously demonstrated in cadaver studies [9]. As a consequence of the ultrasound‐induced melting of the bone cement, local temperatures of up to 80°C are generated, and necrosis of the bone matrix and adjacent soft tissue has been reported [2, 8]. However, the evaluation of postoperative CRP level dynamics revealed no significant change in the systemic inflammatory response between the two study groups. Therefore, it appears that the use of an ultrasonic cement removal device does not exert a relevant effect on systemic inflammation and is not disadvantageous.

One of the most compelling arguments in favour of utilising an ultrasonic cement removal device is its capacity for atraumatic and gentle exposure of the endosteal cortex from bone cement while avoiding cortical perforation. Current literature reports fracture and cortical perforation rates ranging from 2% to 20% when femoral stems are removed using conventional instruments [5, 11, 24]. The limited available studies on the use of ultrasonic cement removal devices indicate notably lower fracture rates of 1%–2% [3]. Our study demonstrated a comparable rate of intraoperative fractures to that reported in the current literature. However, it should be noted that 20 of the examined procedures involved the knee joint, where the fracture risk is lower than in the proximal femur. The observed complication of intraoperative fracture in this study occurred during the explantation of a THA. Due to the small number of cases in the entire series and the limited number of hip revisions, it is not feasible to reliably estimate complication rates. Nevertheless, the study demonstrates that fracture complications cannot be entirely eliminated, even with the utilisation of an atraumatic ultrasonic cement removal device, despite the limited sample size.

This study exhibits several limitations that warrant consideration. Primarily, the retrospective study design introduces inherent constraints regarding data quality and the resultant conclusions. It is imperative to acknowledge that the limited sample size of 22 matched cases restricts the capacity to draw statistically significant conclusions about the study endpoints. Furthermore, the ability to predict complication incidence is compromised due to the evident disproportion between knee and hip joint revisions in this cohort (10:1). Another crucial limitation is the influence of the surgeon's learning curve on the parameters examined. Not only was there a small number of cases, but the learning curve for the ultrasonic cement removal device was probably not yet complete. The 11 patients were also operated on by four senior surgeons, so that the learning curve effect became even more relevant. In particular, the learning curve can have a decisive influence on the operation time and the rate of intraoperative complications.

The paucity of available literature addressing ultrasonically assisted cement removal in arthroplasty revision surgery is noteworthy. Consequently, further prospective investigations are requisite to evaluate, at minimum in the short‐term perspective, whether the utilisation of ultrasonic cement removal systems is associated with reduced infection or re‐infection rates following one‐ and two‐stage arthroplasty revisions.

## CONCLUSION

In summary, the presented data fail to provide substantive evidence supporting the superiority of ultrasound‐guided cement removal over manual techniques in hip and knee revision surgery. Additionally, surgeons must be aware that using ultrasound equipment for cement extraction does not eliminate the risk of fractures occurring during the operation. Based on the data presented in this study, the authors cannot conclude that the use of ultrasound‐guided devices for the removal of cement residues from the medullary canal during revision surgery is superior to conventional techniques. However, additional prospective studies are crucial for a thorough assessment.

## AUTHOR CONTRIBUTIONS

**Marco Haertlé**: Writing–original draft; data curation; formal analysis; visualization. **Lars Tücking**: Formal analysis; writing–review and editing. **Alexander Derksen**: Data curation; writing–review and editing. **Magnus Reulbach**: Writing–review and editing. **Eike Jakubowitz**: Writing–review and editing. **Henning Windhagen**: Writing–review and editing; supervision.

## CONFLICT OF INTEREST STATEMENT

The authors declare no conflicts of interest.

## ETHICS STATEMENT

The study was conducted in accordance with the Declaration of Helsinki and approved by the local Ethics Committee of the Hannover Medical School (code: 11428_BO_K_2024 date: 30.05.2024). Investigation was performed at Hannover Medical School, Department of Orthopaedic surgery, Anna‐von‐Borriesstr. 1‐7, 30625 Hannover, Germany.

## Data Availability

The data sets used and analysed during the current study are available from the corresponding author on reasonable request.
